# Identification of a novel mutation in the cornea specific keratin 12 gene causing Meesmann`s corneal dystrophy in a German family

**Published:** 2010-05-29

**Authors:** Ina Clausen, Gernot I.W. Duncker, Claudia Grünauer-Kloevekorn

**Affiliations:** Department of Ophthalmology, Martin-Luther-University Halle-Wittenberg, Halle (Saale), Germany

## Abstract

**Purpose:**

To report a novel missense mutation of the cornea specific keratin 12 (*KRT12*) gene in two generations of a German family diagnosed with Meesmann`s corneal dystrophy.

**Methods:**

Ophthalmologic examination of the proband and sequencing of keratin 3 (*KRT3*) and *KRT12* of the proband and three other family members were performed. Restriction enzyme analysis was used to confirm the detected mutation in affected individuals of the family.

**Results:**

Slit-lamp biomicroscopy of the proband revealed multiple intraepithelial microcysts comparable to a Meesmann dystrophy phenotype. A novel heterozygous A→G transversion at the first nucleotide position of codon 129 (ATG>GTG, M129V) in exon 1 of *KRT12* was detected in the proband, her two affected sons but not in her unaffected husband or 50 control individuals.

**Conclusions:**

We have identified a novel missense mutation within the highly conserved helix-initiation motif of *KRT12* causing Meesmann`s corneal dystrophy in a German family.

## Introduction

The cornea represents the anterior surface of the eye and must maintain its structural integrity as well as its transparency to retain good vision. The outermost layer of the cornea is presented by the corneal epithelium.

In 1939 the German ophthalmologists Meesmann and Wilke described a dominantly inherited dystrophy affecting the corneal epithelium in three families living in northern Germany [1]. The 34 affected family members showed ocular irritation, tearing, and glare. Slit lamp biomicroscopy revealed myriads of microcystic epithelial opacities extending to the corneal limbus. Twenty-six years after the initial report by Meesmann and Wilke, Behnke and Thiel [2] published an extensive study of the same three northern German families. Kuwabara and Ciccarelli [3] were the first who examined the ultrastructure of the cornea from patients with Meesmann`s corneal dystrophy (MECD; OMIM 122100) in 1964. They were followed by Fine et al. [4] in 1977 and Tremblay and Dube [5] in 1982. The authors described the corneal epithelial basement membrane as thickened with an intracellular “peculiar substance” that reacted with periodic acid and Schiff`s reagent. This substance appeared to be derived from the tonofilaments.

Keratins are a group of water-insoluble proteins that form intermediate filaments in epithelial cells. They can be divided into acidic and basic-neutral subfamilies according to their relative charges, immunoreactivity, and sequence homologies to type I and II wool keratins, respectively. In vivo, a type I keratin (acidic) pairs with a specific type II keratin (basic or neutral) to form a heterodimer [6]. The structural integrity of corneal epithelial cells derives mainly from its unique composition of the cytoskeletal intermediate filaments keratin 3 and keratin 12, whereby disruption of a single keratin polymer can compromise epithelial structure and function [7]. Among keratin proteins the helix-initiation and helix-termination motifs are highly conserved and crucial for intermediate filament heterodimerization.

A breakthrough approached in 1997 when McLean and coworkers [8,9] examined two families from Northern Ireland and German descendants of the family originally described by Meesmann and Wilke. They were able to relate MECD to the keratin cluster on chromosome 17 (17q12–21). In addition, they performed molecular genetic studies by sequencing the candidate genes keratin 3 (*KRT3*) and keratin 12 (*KRT12*) [8,9]. Irvine et al. [8] postulated that dominant-negative mutations in these keratins might be the cause of MECD. Indeed, they found linkage of the disorder to the *KRT12* locus in Meesmann`s original German kindred. Furthermore, in the two pedigrees from Northern Ireland, they found that the disorder cosegregated with *KRT12* in one pedigree and *KRT3* in the other.

**Figure 1:**
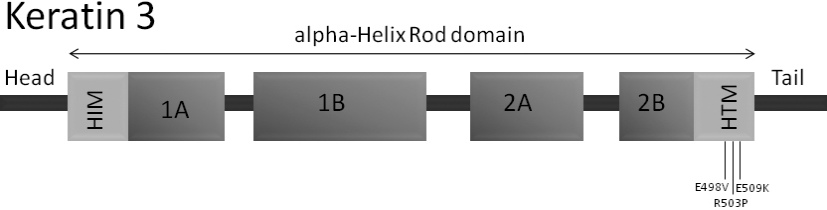
Scheme of the *KRT3* structure. The localizations of the known point mutations within the helix-termination motif are indicated.

**Figure 2:**
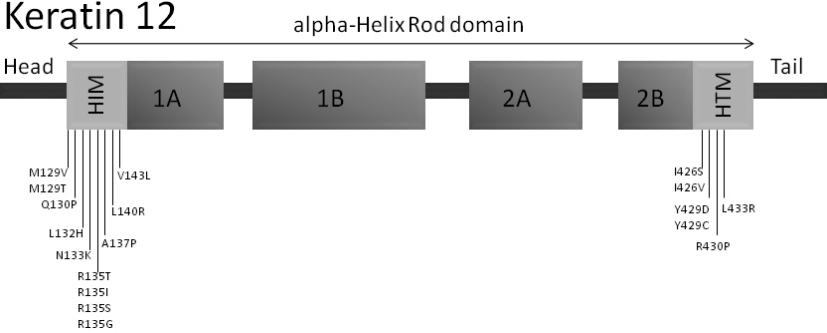
Scheme of the *KRT12* structure. The localizations of the known point mutations within the helix-initiation and the helix-termination motif are indicated.

To our best knowledge three mutations in *KRT3* and 19 mutations in *KRT12* in patients showing MECD have been identified so far (Table 1) [8-22]. In *KRT3* all detected mutations are located in the helix-termination motif (Figure 1), whereas in *KRT12* mutations are found within the helix-initiation and the helix-termination motif as well (Figure 2).

**Table 1:** Review of known mutations in the keratin 3 and keratin 12 genes causing MECD.

**Gene**	**Exon**	**Codon number**	**Codon change**	**Amino acid change**	**References**
*KRT3*	7	498	GAG-GTG	Glu-Val	[10]
	7	503	CGC-CCC	Arg-Pro	[11]
	7	509	GAG-AAG	Glu-Lys	[8]
*KRT12*	1	129	ATG-GTG	Met-Val	Current Report
	1	129	ATG-ACG	Met-Thr	[9]
	1	130	CAA-CCA	Gln-Pro	[12]
	1	132	CTT-CAT	Leu-His	[13]
	1	133	AAT-AAG	Asn-Lys	[14]
	1	135	AGA-ACA	Arg-Thr	[8]
	1	135	AGA-ATA	Arg-Ile	[15]
	1	135	AGA-AGC	Arg-Ser	[16]
	1	135	AGA-GGA	Arg-Gly	[15]
	1	137	GCT-CCT	Ala-Pro	[17]
	1	140	CTG-CGG	Leu-Arg	[15]
	1	143	GTG-CTG	Val-Leu	[8]
	1	143	GTG-TTG	Val-Leu	[18]
	6		1222ins27	400ins9	[16]
	6	426	ATT-AGT	Ile-Ser	[19]
	6	426	ATT-GTT	Ile-Val	[20]
	6	429	TAT-GAC	Tyr-Asp	[15]
	6	429	TAT-TGT	Tyr-Cys	[11]
	6	430	CGC-CCC	Arg-Pro	[21]
	6	433	CTG-CGG	Leu-Arg	[22]

Here we report a novel heterozygous mutation within the helix-initiation motif of *KRT12* causing MECD in three members of a German family.

## Methods

### Subjects

A two generation family with three affected individuals was studied. The index patient (I/2), an 82-year-old woman, was examined by slit-lamp biomicroscopy. Peripheral blood samples of patient I/2, her unaffected husband (I/1) and the two affected sons (II/1, II/2) were obtained. The ophthalmological history of the proband`s deceased parents was unknown. Fifty unrelated volunteers were recruited to serve as controls. All subjects were treated in accordance with the tenets of the Declaration of Helsinki and signed a consent form.

### DNA collection, polymerase chain reaction amplification, and sequencing

Genomic DNA was isolated from peripheral blood leucocytes by standard procedures using the Invisorb Spin Blood Maxi Kit (Invitek, Berlin, Germany). Blood samples (10 ml) treated with EDTA were processed following the manufacturers instructions. Exons 1–9 of *KRT3* and exons 1–8 of *KRT12* were amplified using the primers shown in Table 2. Each PCR reaction was performed in a volume of 50 µl containing 1 µM of forward and reverse primer, 0.5 U of AmpliTaq Gold DNA Polymerase (Applied Biosystems Inc., Foster City, CA), 1 µl dNTP mixture (Applied Biosystems), 5 µl of 10× PCR buffer (Applied Biosystems) and 100 ng of human genomic DNA. Amplification reactions were performed under the following conditions: 5 min of denaturation at 96 °C followed by 35 cycles of denaturation at 96 °C for 30 s, annealing at 60–66 °C for 30 s, extension at 72 °C for 45 s, and a further extension step at 72 °C for 5 min. For direct sequencing, amplified DNA was purified using the QIAquick PCR purification kit (Qiagen GmbH, Hilden, Germany), cycle sequenced with BigDye Terminator v3.1 Cycle Sequencing kit (Perkin Elmer, Waltham, MA) and directly sequenced using an automatic sequencer (ABI 3100 Genetic Analyzer C; Applied Biosystems).

**Table 2:** Primers for polymerase chain reaction direct sequencing of *KRT3* and *KRT12*.

**Exon**	**Forward primer sequence (5`→3′)**	**Reverse primer sequence (5′→3′)**	**Size (bp)**
**Keratin 3**			
1	TGCACAGGTCTTCATTTCCCATCC	TCCTCAACCCTGGATATCTTCCCA	887
2	AGTGTTGCCTGATGTTGCTTCCTG	ACCATGCTTGGAGAAGGAAGGTGA	439
3	ATGGAGGGAGGGAAGAGATGAACT	ATTGCTCCAAAGGCCTGAACTTGG	275
4	GCTCTTTCTTGCTGCAGTTGTGGT	GCACCAGCCTCAAATCTGGAAACA	238
5	AGTGAACAAGCTCCCTCTGTGTTG	TGAAACCTCCAGTGGATCCCGTAA	235
6	AAGGTTTGGTGGGTGATGTTGGAG	ATTTGTGGAGATACTGCCCTGTGG	345
7	AATCCATTGCATGTCAGGAAGGGC	TATCTGGCCCTTGGCCTATGACTT	354
8	TGTTGGTGATGTGCTTTGTGACGG	AAGCCAATCACTTCCCTCTCCTCT	228
9	ACAATAACATAGCAGCTGGCCTGG	AATACTCAGAGGCCCGGAGTGAAA	756
**Keratin 12**			
1a	GGATCCAATTTTGAGTGGAGA	ACCTAGAGAGCCACCTCCTGGGCT	459
1b	AGCCCAGGAGGTGGCTCTCTA	AGTACAGCTAAATTGGAAAAT	369
2	TAGTCTTTTAGGGCTTCAATC	AGGCAGGACAGTAGGACAGA	194
3	GCAAGAAATAGCCCTGAAGA	TTTTGGGTCTGGGAGAAA	297
4	GGCCCAAGAGGACAAAAGTA	GCAGGCCTTTCTGTGAATGT	290
5	AGAAAGGCCTGCGAA	AAAAGAGGAGGGTAGCCAAC	278
6	GTCCCCTCCATCGTTATTTC	CTATTTCTGCTGCCCACTCT	330
7	TCCATTAAAAGCCAGGTTGT	GGTCTTACAGGTTTTGCATT	198
8	GCCTACATTAAACAACCAGT	TGACCAAAATGACTTGTGACTG	370

### Mutation confirmation

The mutation M129V generated a HpyCH4III (New England Biolabs GmbH, Frankfurt, Germany) restriction enzyme site in exon 1b of *KRT12*. Therefore, PCR products of all four family members (three affected and one unaffected individual) as well as 50 unrelated control subjects were submitted to restriction digestion under the following conditions. A reaction mixture containing 10 µl PCR product, 5 µl enzyme, 25 µl NEBuffer 4 (50 mM potassium-acetate, 20 mM Tris-acetate, 10 mM magnesium-acetate, 1 mM dithiothreitol; New England Biolabs), and 170 µl H_2_O was incubated overnight at 37 °C. In the presence of the heterozygous M129V mutation, HpyCH4III digestion resulted in fragments of 293 bp and 76 bp with the additional undigested 369 bp PCR product. The digested products were separated and visualized on a 2% agarose gel stained with ethidium bromide. Digestion analysis was additionally used to screen fifty healthy control subjects for the M129V mutation.

## Results

### Clinical exam

**Figure 3:**
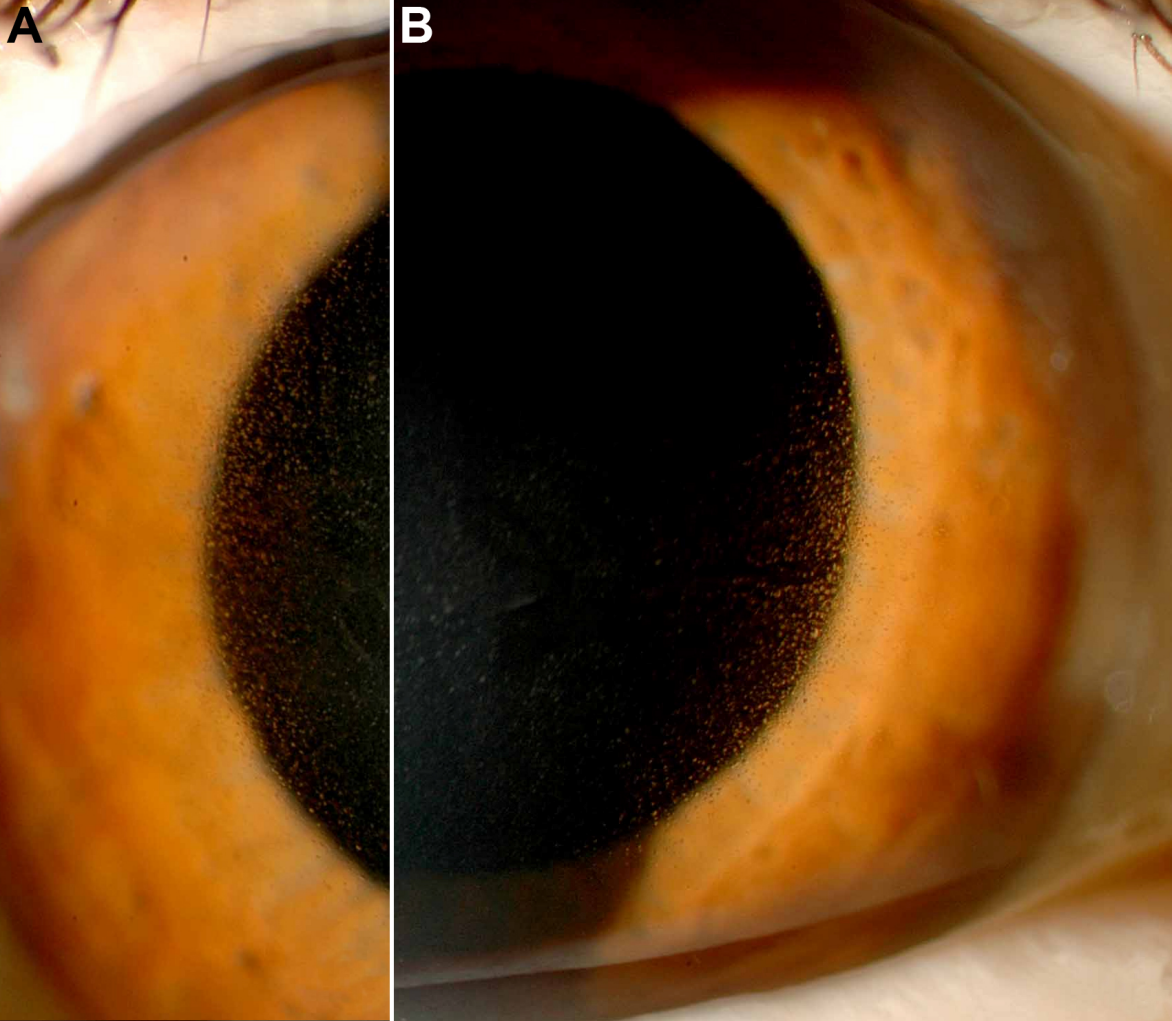
Slit lamp photography of patient I/2. The corneal epithelium showed bilateral myriads of fine cysts predominantly in the center of the cornea (**A**=right eye, **B**=left eye).

Slit lamp examination of patient I/2 revealed multiple microcysts within the corneal epithelium of both eyes with highest density in the center of the cornea (Figure 3). The proband had recurrent corneal erosions, blurred vision, and tearing. Visual acuity was highly reduced (OD finger count, OS 5/20) predominantly due to high myopia with myopic macular degeneration. Additionally, the patient was pseudophacic with posterior chamber lenses after cataract surgery. Ocular tension was within normal range.

### *KRT3* sequence analysis

The sequence analysis of all nine exons of *KRT3* revealed no mutations in the four family members.

### *KRT12* sequence analysis

**Figure 4:**
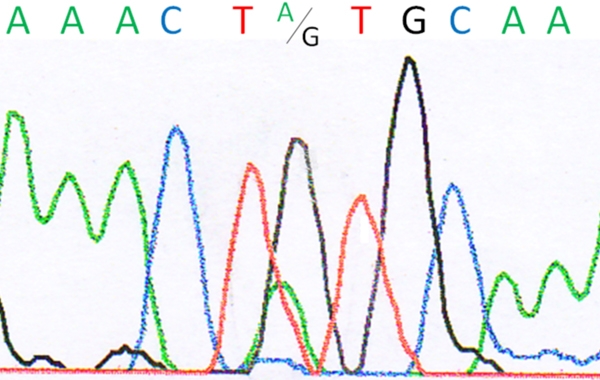
Chromatogram of the heterozygous 409G>A mutation at codon 129 in the keratin 12 gene of patient I/2.

**Figure 5:**
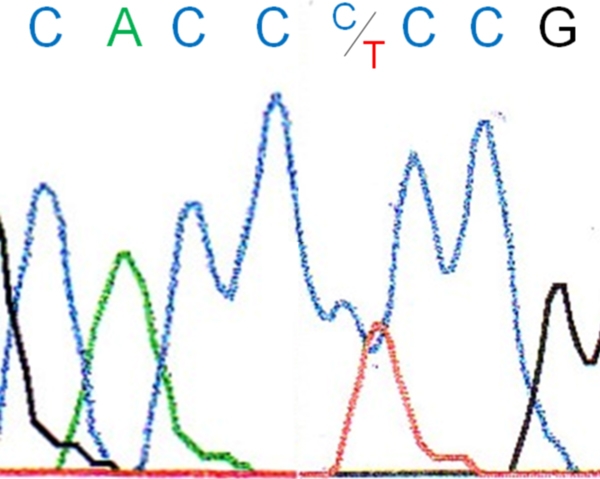
Chromatogram of the single point polymorphism 67C>T in the keratin 12 gene of patient I/2.

Direct forward and backward sequencing of exon 1b of *KRT12* disclosed the heterozygous 409A>G transversion at the first nucleotide position of codon 129 (ATG>GTG) in the proband I/2 (Figure 4) and her two affected sons (II/1, II/2). This is predicted to substitute a valine residue for methionine (M129V, NM_000223). This missense mutation was absent in the unaffected husband (I/1). Additionally, the proband and her sons harbored the single-nucleotide polymorphism (SNP) 43C>T (Figure 5), resulting in an amino acid change in codon 15 from proline to serine (P15S, NM_000223). Further sequence analysis of exons 2–8 did not show any alterations.

### Mutation confirmation

**Figure 6:**
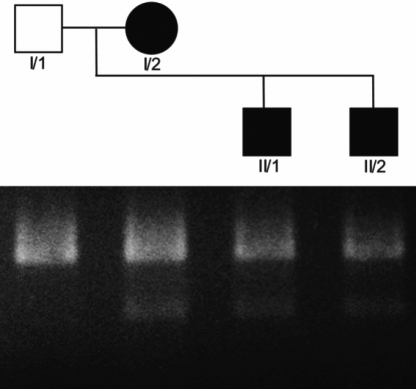
Pedigree of the examined German family. Below results of restriction enzyme analysis after HpyCH4III digestion on a 2% agarose gel stained with ethidium bromide. In the presence of the 409G>A mutation the full PCR fragment of 369 bp is cut into bands of 293 and 76 bp (the latter not shown) as seen in the proband (I/2) and her two affected sons (II/1, II/2). Undigested DNA was seen in the unaffected husband (I/1).

Restriction enzyme analysis employing HpyCH4III resulted in digested fragments of 76 bp and 293 bp in the proband (I/2) and her two affected sons (II/1, II/2) as shown in Figure 6. Since the found mutation is heterozygous, the undigested wild type fragment of 369 bp is also seen. Exclusively undigested wild type DNA was seen in the unaffected husband (I/1) and in fifty healthy control individuals (data not shown).

## Discussion

All known mutations in the cornea specific genes *KRT3* and *KRT12* are responsible for the development of Meesmann`s corneal dystrophy, an autosomal dominant disorder of the corneal epithelium. Until the present study, three mutations have been reported in *KRT3* and 19 in *KRT12*, including a 27-nucleotide long insertion in exon 6. All mutations are summarized in Table 1. It is noteworthy, that the majority of the *KRT12* mutations are localized within the region encoding the helix-initiation motif. In contrast, the known mutations of *KRT3* are exclusively within the helix-termination motif. In keratin proteins, the helix-initiation and –termination motifs are highly conserved regions playing an important role in normal filament assembly [23].

With the predicted amino acid substitution M129V we revealed the third mutation of *KRT12* in a German family. Previously, Irvine and coworkers [8] identified the point mutation R135T in descendants of the German kindred originally described by Meesmann and Wilke [1]. In 2000, Corden et al. [12] reported a Q130P substitution in a southern German family.

Another nucleotide exchange within the same codon (M129T) predicting the substitution of the methionine (ATG) residue by threonine (ACG) was described independently by Cordon et al. [9] and Nichini et al. [19] in American families. It is unknown whether these individuals are related.

Our index patient, an 82-year-old female, had a rather severe case of MECD with photophobia, tearing, and recurrent painful corneal erosions since adulthood. Visual acuity was highly reduced due to myopic macular degeneration. Additionally, the proband and her affected sons harboured the SNP 67C>T predicting the amino acid substitution P15S. This SNP has been previously described by Nielsen et al. [18] in an asymptomatic Danish family. The allele frequency in healthy probands was calculated to be 29%. Since the affected family members described by Nielsen et al. [18] presented an asymptomatic form of MECD, it seems unlikely, that the additional SNP should be responsible for the severity of symptoms in our patient.

Still, it seems cryptic why certain mutations lead to a more or less severe phenotype. Severe cases of MECD have been reported due to mutations within the helix initiation motif [12,14] as well as the helix termination motif [21]. Furthermore, the quality of the amino acid change seems not to elucidate the genotype-phenotype correlation. The V143L substitution in an asymptomatic Danish family means the exchange of two neutral aliphatic amino acids [18]. However, in our patient the aliphatic neutral methionine is replaced by another aliphatic and neutral amino acid (valine) as well.

Moreover, mutations in *KRT3* cause a MECD phenotype indistinguishable from *KRT12* mutations. Kao et al. [7] demonstrated the importance of functional *KRT3*/*KRT12* heterodimers for corneal stability in a *KRT12* knockout mouse model. Any modification that results in an impairment of the heterodimerization process will lead to corneal fragility. This could explain why mutations in any member of the heterodimer lead to almost identical phenotypes. Therefore, other individual factors may contribute to the MECD phenotype.

The present study adds another novel mutation to the collection of mutations causing Meesmann`s corneal dystrophy.
